# Iterative X-ray spectroscopic ptychography^1^

**DOI:** 10.1107/S1600576720006354

**Published:** 2020-07-08

**Authors:** Huibin Chang, Ziqin Rong, Pablo Enfedaque, Stefano Marchesini

**Affiliations:** a School of Mathematical Sciences, Tianjin Normal University, Tianjin, People’s Republic of China; b Computational Research Division, Lawrence Berkeley National Laboratory, Berkeley, CA, USA

**Keywords:** spectromicroscopy, ptychography

## Abstract

Spectroscopic ptychography is a powerful technique to determine the chemical composition of a sample with high spatial resolution. In spectro-ptychography, a sample is rastered through a focused X-ray beam with varying photon energy so that a series of phaseless diffraction data are recorded. Each chemical component in the material under investigation has a characteristic absorption and phase contrast as a function of photon energy. Using a dictionary formed by the set of contrast functions of each energy for each chemical component, it is possible to obtain the chemical composition of the material from high-resolution multi-spectral images. This paper presents SPA (spectroscopic ptychography with alternating direction method of multipliers), a novel algorithm to iteratively solve the spectroscopic blind ptychography problem. First, a nonlinear spectro-ptychography model based on Poisson maximum likelihood is designed, and then the proposed method is constructed on the basis of fast iterative splitting operators. SPA can be used to retrieve spectral contrast when considering either a known or an incomplete (partially known) dictionary of reference spectra. By coupling the redundancy across different spectral measurements, the proposed algorithm can achieve higher reconstruction quality when compared with standard state-of-the-art two-step methods. It is demonstrated how SPA can recover accurate chemical maps from Poisson-noised measurements, and its enhanced robustness when reconstructing reduced-redundancy ptychography data using large scanning step sizes is shown.

## Abstract 2

Spectroscopic ptychography is a powerful technique to determine the chemical composition of a sample with high spatial resolution. This paper presents a novel algorithm to iteratively solve the spectroscopic blind ptychography problem.

## 1. Introduction   

X-ray spectro-microscopy is a powerful technique to study the chemical and morphological structure of a material at high resolution. The contrast of the material under study is recorded as a function of photon energy, and this spectral absorption contrast can later be used to reveal details about its chemical, orbital or magnetic state (Stöhr, 2013 ▸; Koningsberger & Prins, 1988 ▸). The idea is that, because different chemical components interact differently with the beam at different energies, the composition map of a sample can be solved by using measured reference spectra (a dictionary).

Compared with standard lens-based microscopy, X-ray ptychography can provide much finer spatial resolution, while also providing additional phase contrast of the sample (Nellist *et al.*, 1995 ▸; Chapman, 1996 ▸; Rodenburg & Faulkner, 2004 ▸; Rodenburg *et al.*, 2007 ▸). Ptychography is based on retrieving the phase of diffraction data recorded to a numerical aperture that is far larger than what X-ray optics can technically achieve. In ptychography, the probe (illumination) is almost never completely known, so a joint recovery problem (sample and probe) is typically considered, referred to as blind ptychography. Several algorithms to solve both standard and blind ptychography problems have been published in the literature, which also consider a variety of additional experimental challenges (Maiden & Rodenburg, 2009 ▸; Thibault *et al.*, 2009 ▸; Thibault & Guizar-Sicairos, 2012 ▸; Wen *et al.*, 2012 ▸; Marchesini *et al.*, 2013 ▸; Horstmeyer *et al.*, 2015 ▸; Hesse *et al.*, 2015 ▸; Odstrci *et al.*, 2018 ▸; Chang *et al.*, 2019*a* ▸).

As in standard spectro-microscopy, it is possible to perform spectroscopic ptychography by recording diffraction data at different X-ray photon energies. In recent years, spectro-ptychography has become an increasingly popular chemical analysis technique (Beckers *et al.*, 2011 ▸; Maiden *et al.*, 2013 ▸; Hoppe *et al.*, 2013 ▸; Shapiro *et al.*, 2014 ▸; Farmand *et al.*, 2017 ▸; Shi *et al.*, 2016 ▸). However, the standard methodology involves independent ptychographic reconstructions for each energy, followed by component analysis, *i.e.* spectral imaging analysis based on a known reference spectrum or multivariate analysis (Adams *et al.*, 1986 ▸; Lerotic *et al.*, 2004 ▸; Shapiro *et al.*, 2014 ▸; Yu *et al.*, 2018 ▸). More recently, a low-rank constraint (Vaswani *et al.*, 2017 ▸) for multi-channel samples was proposed, together with a gradient descent algorithm with spectral initialization to recover the higher-dimension phase-retrieval problem (without component analysis). Other work proposed a hierarchical model with a Gaussian–Wishart hierarchical prior and developed a variational expectation–maximization algorithm (Liu *et al.*, 2019 ▸). Also, a matrix-decomposition-based low-rank prior (Chen *et al.*, 2018 ▸) has been exploited to reconstruct dynamic time-varying targets in Fourier ptychographic imaging.

In this paper we propose a novel technique to solve the blind X-ray spectro-ptychography problem, based on coupling the diffraction data from each photon energy and iteratively retrieving the chemical map of the sample. The proposed algorithm, referred to as SPA (spectroscopic ptychography with ADMM), works with both completely and partially known reference spectra. The method is designed using the alternating direction method of multipliers (ADMM)  (Glowinski & Tallec, 1989 ▸; Chang *et al.*, 2019*a* ▸) framework, employing also total variation (TV) regularization (Rudin *et al.*, 1992 ▸) on the chemical map. Compared with the standard two-step methods, the proposed joint reconstruction algorithm can generate much higher quality results without presenting the phase ambiguity problem inherent to two-step methods.^2^ The simulation analysis shows the efficient convergence ratio of SPA and demonstrates the increased robustness of the method to large step sizes, being able to retrieve features lost when using standard two-step methods. The algorithm is described and analyzed with and without TV regularization for both partially and completely known dictionary cases.

## 2. Spectroscopic ptychography model   

The main operators used in this section are given in Table 1 ▸.

Given *L* different energies of X-rays going through a sample illuminated by a probe 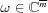, a collection of phaseless intensities 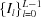 are measured in the far field, such that with Poisson fluctuation caused by photon counting we have
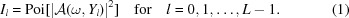
Here 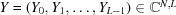 is the sample contrast map for each X-ray energy, 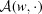 is the forward operator for ptychography for a given probe *w*, Poi denotes the Poisson-noise contamination, and the notations 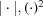 denote the pointwise absolute and square values of the vector, respectively. Note that the probe ω and each column of contrast maps 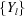 are all 2D images, written as vectors by a lexicographical order. The relationship between the contrast map 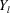 observed by ptychography at each energy and an unknown sample elemental map *X* made of *C* elements is governed by the spectral contrast of each element, stored in a ‘dictionary’ *D* of known values.

Specifically, following similar notation to Chang, Enfedaque *et al.* (2018 ▸), the bilinear operator 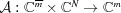 is defined as 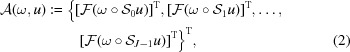
where 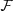 denotes the discrete Fourier transform, 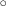 denotes the Hadamard product (pointwise multiplication) of two vectors, and 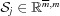 is a binary matrix that defines a small window with the index *j* and size 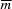 over the entire image *u* (taking small patches out of the entire image).

For different energies, assuming that a spectrum dictionary 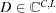 (or its absorption part) is measured in advance, having *C* components for different materials or particles, and given a sufficiently thin specimen, the sample contrast maps can be approximated by first-order Taylor expansion 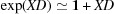 3 as 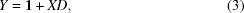
with 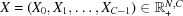 being the elemental thickness map of the sample (each column of the thickness map denotes the thickness of each component in the object).

To determine the thickness map *X*, with a completely known spectrum *D*, one has to solve the following problem: 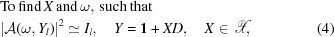
with non-negative thickness constraint set 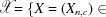 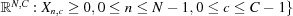. Letting the illumination be normalized, *i.e.* 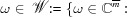 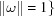, the total variation regularized nonlinear optimization model can be established by assuming the piecewise smoothness of the thickness map as
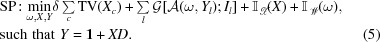


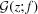
 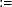 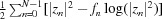 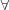 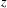 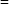 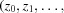 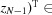 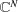, 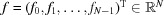, derived from the maximum likelihood estimate of Poisson-noised data (Chang, Lou *et al.*, 2018 ▸), 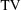 denotes the standard total variation semi-norm (Rudin *et al.*, 1992 ▸) to enforce the piecewise smooth structure of 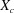 [the *c*th column of the mixing matrix (thickness map) defined in equation (3)], and δ is a positive constant to balance the regularization and fitting terms (larger δ produces stronger smoothness). 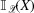, 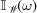 denote the indicator functions, with 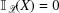 if 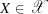; 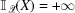 otherwise. We remark that this is a convenient way to enforce hard constraints within an optimization formulation.

Experimentally, only the real-valued part (absorption) of the dictionary 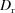 is measured. As *X* is real valued, we consider the following relation: 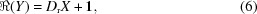
where 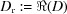. Similarly, we derive the following for spectroscopic ptychography with an incomplete dictionary (SPi):
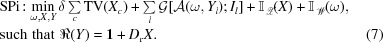

*Remark*: Rather than solving the ptychography imaging independently for each energy, we use the low-rank structure of the recovery results of different energies,* i.e.* the rank of the matrix 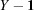 is no greater than that of *X*.

## 3. Proposed iterative algorithm   

ADMM (Glowinski & Tallec, 1989 ▸) is a powerful and flexible tool that has already been applied to both ptychography (Wen *et al.*, 2012 ▸; Chang *et al.*, 2019*a* ▸) and phase tomography problems (Chang *et al.*, 2019*b* ▸; Aslan *et al.*, 2019 ▸). In this work we also adopt the ADMM framework to design an iterative joint spectro-ptychography solution. We construct the proposed algorithm considering both complete and incomplete dictionary cases.

### 3.1. Complete dictionary   

On the basis of the spectro-ptychography model [equation (5)] for a complete dictionary of spectra, we design the proposed SPA algorithm as described below.

Let 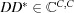 be non-singular, where 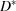 denotes the Hermitian matrix of *D*, *i.e.* 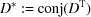. Considering the constraint in equation (3), the following equivalent form can be derived:
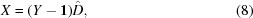
with 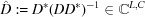. Accordingly, the following equivalent model can be considered, by introducing auxiliary variables 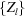: 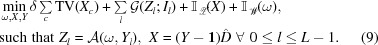
The benefits of considering equation (8) instead of equation (3) lie in the fact that (i) the multiplier will be a low-dimensional variable, since the dimension of *Y* is much higher than that of *X*, and (ii) the subproblem with respect to the variable *X* can be more easily solved.

An equivalent saddle point problem for equation (9), based on the augmented Lagrangian, can be derived as
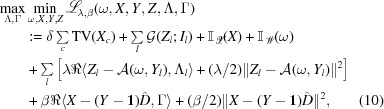
with the multipliers 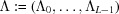 and Γ, where 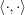 denotes the inner product of two vectors (or trace norms for two matrices).

The above saddle point problem can be solved by alternating minimization and update of the multipliers. We first define each sub-minimization problem. The ω subproblem, with the additional proximal term, can be expressed as 
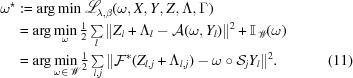
The first-order gradient of the above least-squares problem (without constraint) is given as 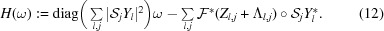
Consequently, the projected gradient descent scheme with preconditioning can be derived as 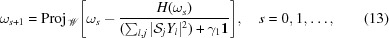
with parameter 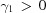 in order to avoid division by zeros, and 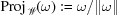. Here the parameter 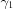 is heuristically set to be a small scalar related to the maximum value of 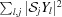, *e.g*. 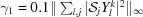.

The *X* subproblem can be expressed as
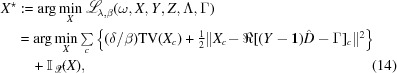
where 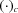 denotes the *c*th column of a matrix. Since it is common practice to solve the total variation denoising problem by using a first-order operator-splitting algorithm (Wu *et al.*, 2011 ▸; Chambolle & Pock, 2011 ▸), we directly give the approximate solution below: 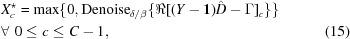
with 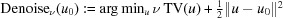. Here we remark that, to seek the exact solution with this positivity constraint, one may need more auxiliary variables and inner loops (Chan *et al.*, 2013 ▸). For simplicity, we did not exactly solve the constraint problem, and instead, the above approximation is derived by the standard TV-L2 denoising without constraint and then a projection to the positivity constraint set.

The *Y* subproblem, with additional proximal term 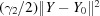 and previous iterative solution 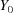, is expressed as 
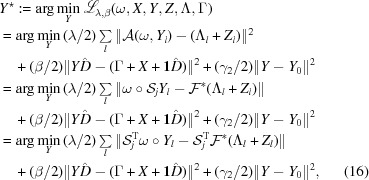
where 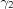 is a positive scalar similar to the parameter 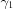.

By calculating the first-order gradient of the above least-squares problem, one has
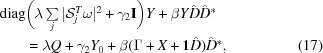
with identity operator 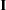, where 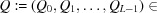 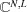 with 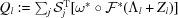. Equation (17) is actually the Sylvester equation (Sylvester, 1884 ▸; Simoncini, 2016 ▸).

Assuming that the positive Hermitian 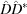 has the singular value decomposition (SVD) 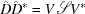, with diagonal matrix (diagonal elements are singular values) 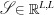 and unitary matrix 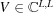, and by introducing 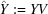, we derive
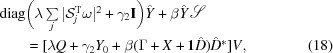
such that the closed-form solution can be expressed as 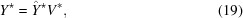
where 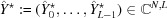 and 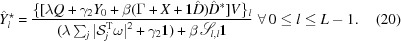
For the 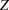 subproblem, we have (Chang, Lou *et al.*, 2018 ▸) 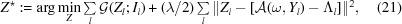
which gives 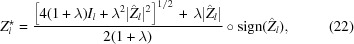
with 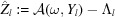. The above calculations and the update of the multipliers form the basis of the baseline SPA algorithm, summarized in Appendix *A* [App appa].

### 3.2. Incomplete dictionary   

A complete dictionary is often difficult to obtain without an independent experiment prior to a spectro-ptychography experiment. The material’s components and their chemical states are often not known in advance. Moreover, the real part of the refractive index component is often not well known (Henke *et al.*, 1993 ▸). It is more difficult to measure because it requires interferometric or reflectometry measurements rather than simple absorption spectroscopy measurements, and reflectometry experiments are less commonly done. While the Kramers–Kronig relationships relate real and imaginary parts, the relationship requires a spectral measurement from 0 to infinity, which is not possible to measure in finite time. Standard techniques to extend absorption spectra can only produce approximate values in the imaginary component. Hence, it is attractive in practice to provide a version working with the real part only.

In this subsection, we propose a variation of the SPA algorithm to solve the joint spectro-ptychography problem when the dictionary of spectra is only partially know, based on the model proposed in equation (7). By assuming that 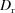 has full row rank, *i.e.* 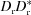 is non-singular, with 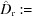 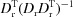 known in advance, we have
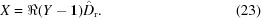
Consequently, the following equivalent problem can be solved instead of equation (7): 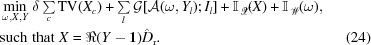
Similarly to the previous subsection, introducing the multiplier 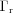 and auxiliary variable *Z* yields the saddle point problem below, with the help of the augmented Lagrangian of equation (24):
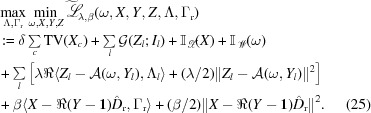
Below, we focus only on the differences with respect to Algorithm 1 (Appendix *A* [App appa]). For the *X* subproblem, we have
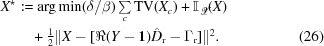
Hence we get 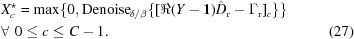



For the *Y* subproblem with proximal terms 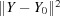, we have
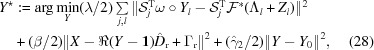
which results in the following equations with respect to the real and imaginary parts, respectively: 
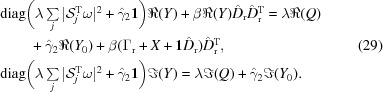
Then, the real part of *Y* can be solved by equations (19) and (20), while the imaginary part can be simply computed by
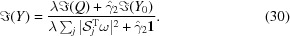



The overall SPA algorithm with an incomplete dictionary is summarized in Appendix *B* [App appb].

## 4. Simulation and reconstruction results   

**Figure 1:**
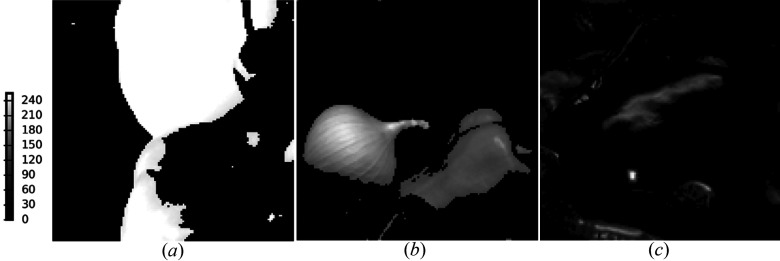
Truth for the three different materials: (*a*) PMMA, (*b*) PS and (*c*) constant.

**Figure 2:**
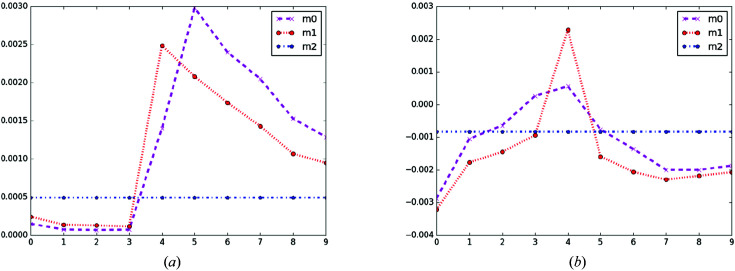
Spectrum dictionaries [three different materials m0 (PMMA), m1 (PS) and m2 (constant)], with real part (*a*) and imaginary part (*b*). The *x* and *y* axes denote the order of the ten spectra and different energies, respectively.

In the simulation analysis of the proposed algorithms we consider the synthetic thickness maps of three different materials, extracted from three (RGB) channels of a natural color image (after thresholding and shift, consisting of 256 × 256 pixels), shown in Fig. 1 ▸. The real part of the spectrum dictionary [for two different materials, (*a*) PMMA (polymethyl methacrylate) and (*b*) PS (polystyrene), plus (*c*) a constant with respect to ten different energies] was measured at the Advanced Light Source (Yan *et al.*, 2013 ▸), and the imaginary part was derived using the Kramers–Kronig relations (Kronig, 1926 ▸). Both real and imaginary part dictionaries are shown in Fig. 2 ▸.

**Figure 3:**
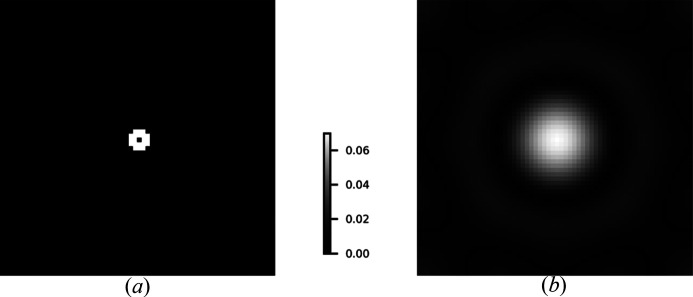
(*a*) Lens (binary) and (*b*) probe (64 × 64 pixels).

The ptychography measurements are simulated with Poisson noise contamination, using a single grid scan at each energy. A standard zone plate with annular shape diffracts an illumination (Fig. 3*a* ▸) onto the sample after the focused probe (Fig. 3*b* ▸) has gone through an order-sorting aperture. The zone plate annular aperture is mapped onto the detector by geometric magnification as ‘outer diameter’ (in µm) and corresponds to an annular ring on the detector of dimension (outer diameter/detector pixel size) × (detector distance/focal distance). The illumination probe [Fig. 3 ▸(*b*)] has a beam width (FWHM) of 16 pixels. The relationship between pixels and actual dimensions in the far-field approximation is as follows: illumination pixel (real-space) dimensions = (wavelength × detector distance)/(detector number of pixels × detector pixel size). The zone plate’s distance from the sample is assumed to be adjusted proportionally with energy to keep the sample in focus, as is usually done experimentally. We also assume that the detector distance is adjusted to maintain the spatial frequencies on the same detector pixels.

In order to evaluate the recovered results, the signal-to-noise ratio (SNR) in dB is used, which is defined below: 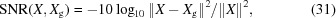
where 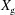 corresponds to the ground-truth thickness.

We compare the proposed iterative SPA algorithm with the standard two-step method. The two-step method consists of (i) performing ptychography reconstruction using a joint illumination, then (ii) performing spectroscopy analysis with a known dictionary (or known real part), and finally (iii) correcting the phase ambiguity for different energies.

When assessing the performance of SPA, we consider both with and without regularization cases, where we simply set the regularization parameter 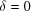 and slightly adjust the algorithm by replacing Step 2 with 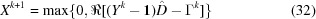
for baseline SPA and 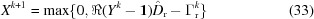
for the incomplete dictionary case.

### 4.1. Reconstruction quality   

**Figure 4:**
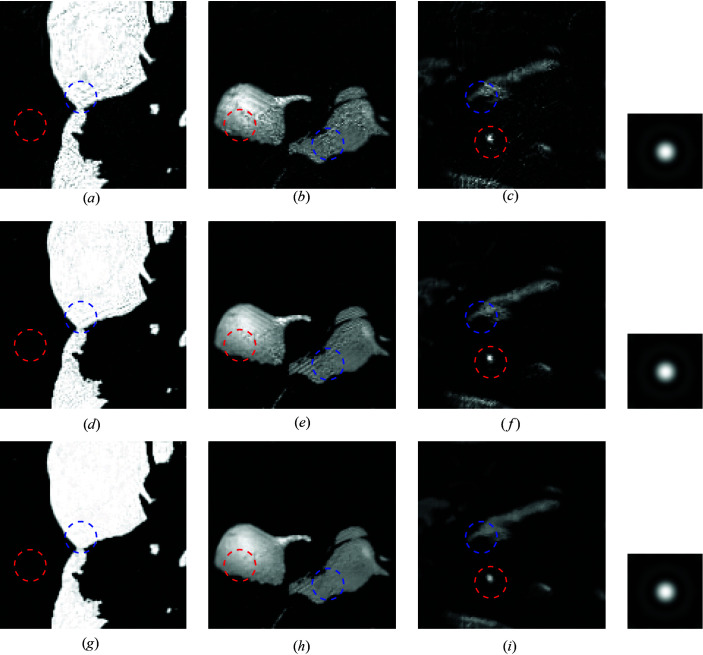
Reconstruction results using a known dictionary of spectra from Poisson-noised data with SNR = 29.2 dB and scan step size = 32. (*a*)–(*c*) Standard two-step method; (*d*)–(*f*) SPA without regularization; (*g*)–(*i*) SPA with TV regularization. The recovered probes are shown in the right column for the two-step method, SPA and SPA with TV (from top to bottom).

**Figure 5:**
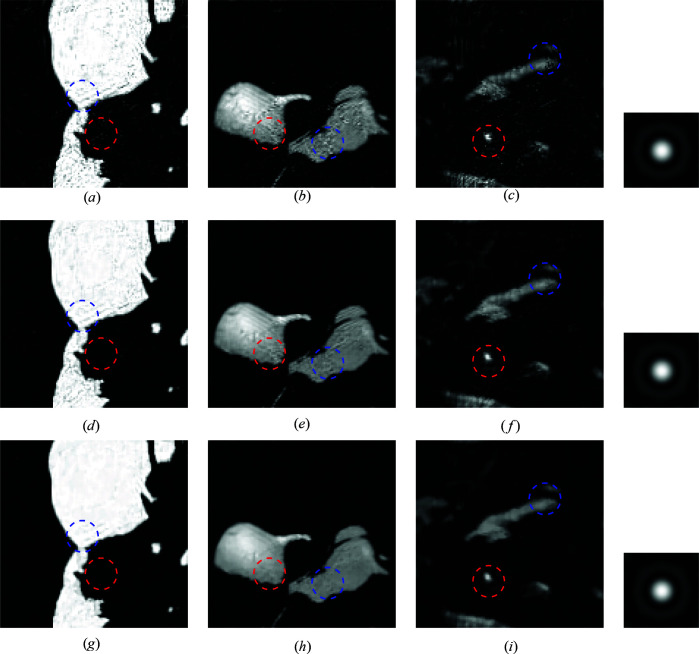
Reconstruction results using a partially known dictionary of spectra from Poisson-noised data with SNR = 29.2 dB and scan step size = 32. (*a*)–(*c*) Standard two-step method; (*d*)–(*f*) SPA without regularization; (*g*)–(*i*) SPA with TV regularization. The recovered probes are shown in the right column for the two-step method, SPA and SPA with TV (from top to bottom).

The first simulation assesses the reconstruction quality achieved by the proposed SPA algorithm, compared with the two-step method, when using a scan step size of 32 pixels. Figs. 4 ▸ and 5 ▸ depict the reconstructed images when using complete and incomplete dictionaries, respectively. The SPA simulations are performed without and with regularization in rows 2 and 3, respectively, of Figs. 4 ▸ and 5 ▸. Visually, we can see obvious artifacts in the recovered images when using the two-step method (first row of Figs. 4 ▸ and 5 ▸). Such artifacts are greatly enhanced when the image is reconstructed using SPA. Specifically, clear improvements can be identified in the regions corresponding to the red and blue circles for all three materials in both Fig. 4 ▸ and Fig. 5 ▸. The SNRs of the recovery results parallel the visual analysis. For the completely known dictionary, the two-step method achieves an SNR of 14.0 dB for the above simulation, whereas SPA achieves 18.1 dB (no regularization) and 18.8 dB (regularization). In the partially known dictionary simulation, the SNRs are 13.8, 15.8 and 16.7 dB, for the two-step method and SPA with no regularization and regularization, respectively, which achieves a comparative gain of more than 2 dB, similarly to the known dictionary case.

The phase ambiguity is an inherent problem of the two-step method that causes a loss in reconstruction accuracy. For example, for the simulation shown in Fig. 4 ▸, the SNR without phase correction is only 12.3 dB, reaching 14.0 dB after applying correction. Even when using an effective phase correction post-process, SPA proves to be more efficient for the simulations performed: higher-quality reconstructions are achieved overall, and there is no need to correct the phase ambiguity because of the iterative reconstruction exploiting the low-rank structure and positivity constraint of the thickness function.

### 4.2. Robustness and convergence   

**Figure 6:**
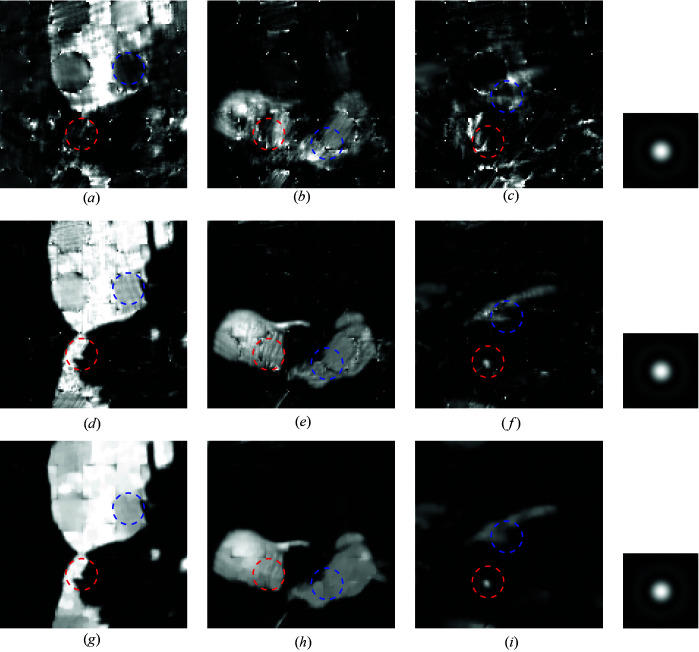
Reconstruction results using a known dictionary of spectra from Poisson-noised data with SNR = 29.0 dB and scan step size = 40. (*a*)–(*c*) Standard two-step method; (*d*)–(*f*) SPA without regularization; (*g*)–(*i*) SPA with TV regularization. The recovered probes are shown in the right column for the two-step method, SPA and SPA with TV (from top to bottom).

The following simulation assesses the robustness of the proposed algorithm when varying the scanning step sizes. The quantitative results of this simulation are presented in Table 2 ▸. The results demonstrate the enhanced robustness of SPA when handling larger step sizes, compared with the reference two-step method, achieving up to 10 dB increase in SNR. To permit a better visual analysis, we provide the reconstruction results of the three algorithms with 40 pixels step size in Fig. 6 ▸. The figure highlights the dramatic improvement achieved by SPA compared with the standard two-step method when reconstructing low-redundancy ptychographic data. Specifically, we can see how the features within the blue and red circles are almost lost in the two-step reconstruction, while they can be clearly observed when reconstructing using SPA.

**Figure 7:**
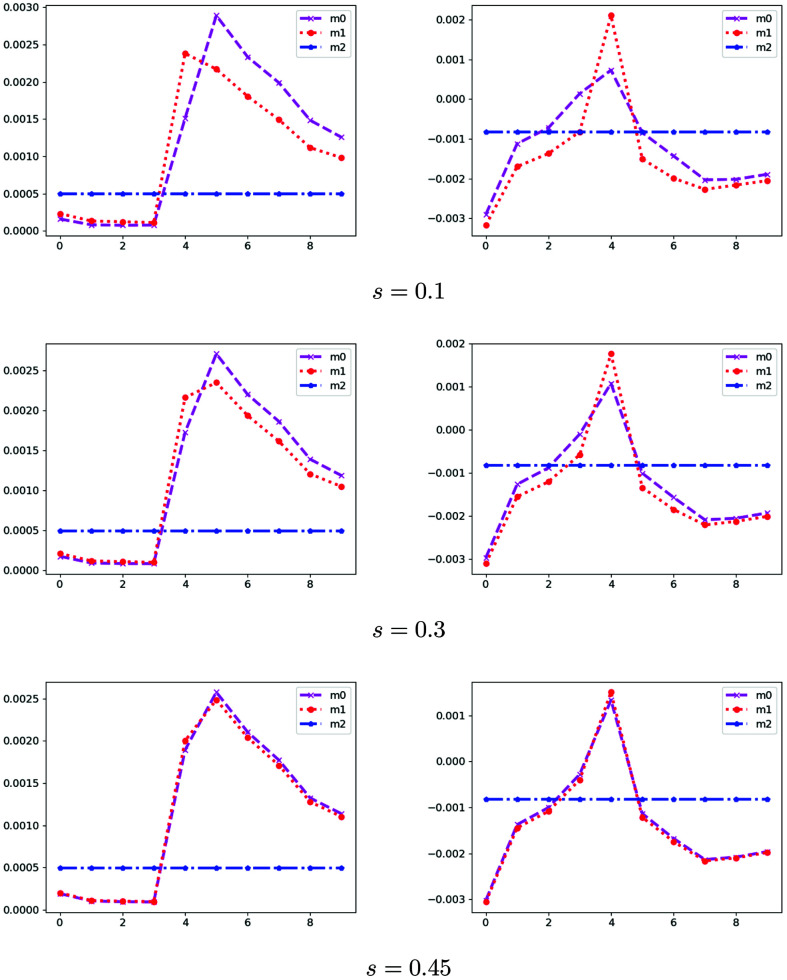
Synthetic spectrum dictionaries 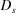 for different *s* [three different materials m0 (PMMA), m1 (PS) and m2 (constant)], for real part (left) and imaginary part (right). The *x* and *y* axes denote the spectrum and different energies, respectively.

**Figure 8:**
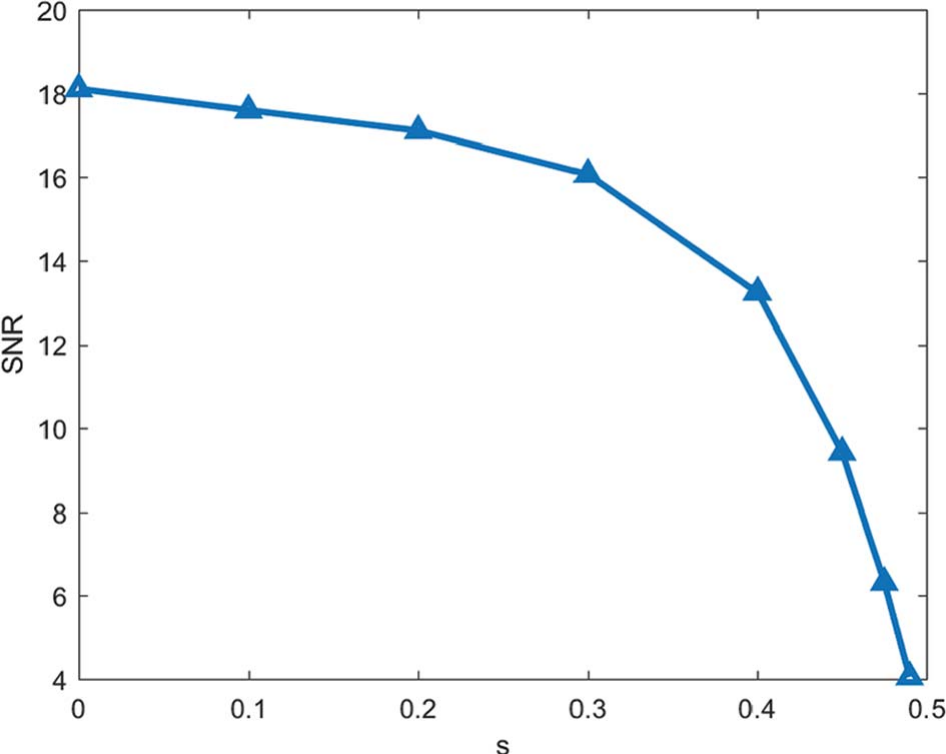
SNR changes versus similarity parameter *s*.

**Figure 9:**
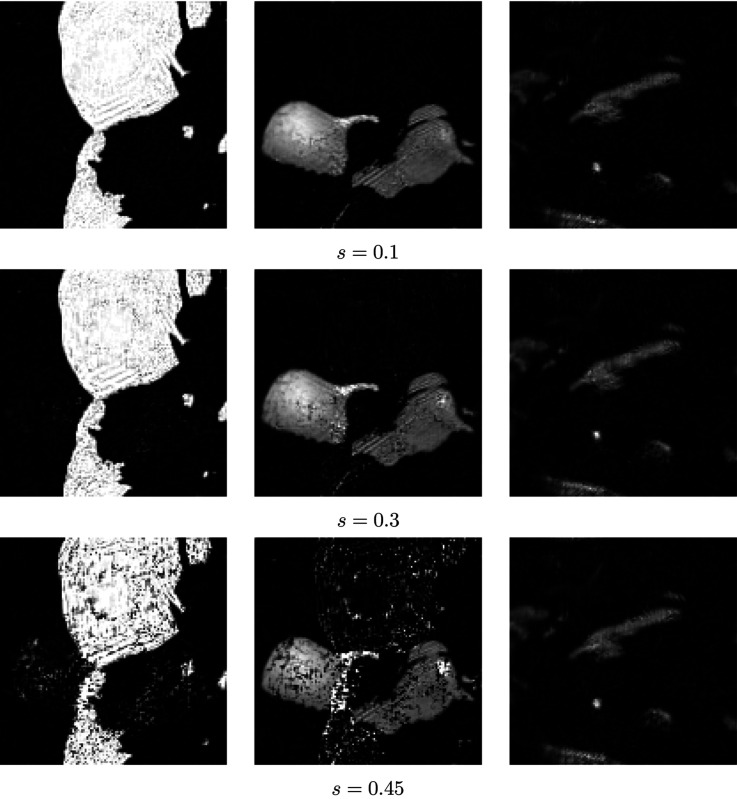
Reconstruction results by proposed SPA using different dictionaries of spectra (*s* = 0.1, 0.3, 0.45), from Poisson-noised data with SNR = 29.2 dB and scan step size = 32.

Generally speaking, to make the proposed algorithms work, the basic condition is to assume *D* has full row rank such that 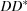 is non-singular. However, the performance should also rely on the similarity of spectral elements. Here we introduce a factor 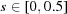 to generate a new dictionary 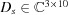 with 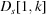 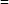 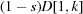 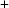 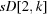, 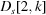 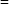 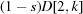 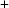 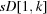 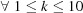. We know that (1) 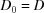 and (2) the first two rows are exactly the same if 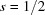 (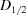 does not have full row rank). (See Fig. 7 ▸ for the dictionaries with *s* = 0.1, 0.3 and 0.45.) Therefore, the parameter *s* can be used to control the similarity of the new spectral dictionary (larger *s* implies higher similarity). We test the impact of the proposed SPA algorithm by the different similarity of spectral dictionaries (see the SNR changes in Fig. 8 ▸ with *s* 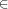 {0, 0.1, 0.2, 0.3, 0.4, 0.45, 0.475, 0.49} and the reconstruction results in Fig. 9 ▸ with *s* = 0.1, 0.3, 0.45). We also know that the quality of reconstruction results obtained by the proposed SPA decays as the spectral dictionaries become similar (the parameter *s* gets close to 0.5). Hence, to get a better reconstruction, one should design the experiments with little similarity in spectral dictionaries.

**Figure 10:**
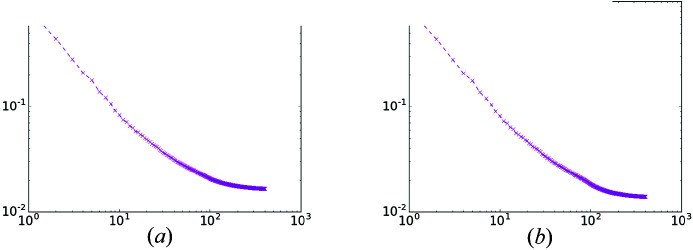
Error 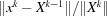 variation versus iteration number for SPA without (*a*) and with TV regularization (*b*). The *x* and *y* axes denote iteration numbers and errors, respectively.

The last simulation depicts the error curve achieved by the SPA algorithm, shown in Fig. 10 ▸. The results demonstrate a steady decrease of the successive errors of the proposed algorithm, both with and without regularization.

## 5. Conclusions   

This paper presents the first iterative spectroscopy ptychography solution. The proposed SPA algorithm is based on a novel spectro-ptychography model and it is constructed considering both a completely known and a partially known dictionary. Numerical simulations show that SPA produces more accurate results with clearer features compared with the standard two-step method. In the future, we will extend our work to thicker samples, where the first-order Taylor expansion is not sufficiently accurate. We also plan to investigate the use of Kramers–Kronig relationships (Hirose *et al.*, 2017 ▸), explore the case using a completely unknown dictionary and further provide software for real experimental data analysis.

## Figures and Tables

**Table 1:** Main variables and operators defined in Section 2

Notations	Explanations
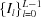	Measured intensities
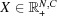	Elemental thickness maps of the sample
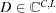	Spectrum dictionary
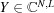	Sample spectral contrast maps
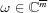	Probe
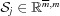	Binary matrix to take image patches
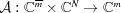	Forward operator for ptychography
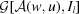	Poisson likelihood estimation
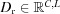	Real part of spectrum dictionary
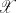	Non-negative thickness constraint
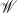	Normalized constraint of the probe
TV	Total variation regularization

**Table 2:** SNR in dB from reconstruction results with different scan step sizes when using the two-step method, SPA and SPA with TV regularization

Step size		36	38	40
SNR	Two-step	11.5	7.0	3.4
	SPA	15.1	15.5	12.3
	SPA + TV	16.0	16.0	13.8

## Footnotes

2 In the first step of ptychography reconstruction for the two-step method, each sample contrast map is recovered independently, which results in different phase factors for different maps.

3
 denotes the matrix with all elements being one, of the same size as *Y*.  denotes the pointwise exponentiation of the matrix.

## Appendix A Algorithm 1: SPA

(0) Initialization: compute the SVD of  as . Set , , , ,  and . Set .

(1) Update the probe  by the one-step projected gradient descent method:

with .

(2) Update the thickness function  by

(3) Update  by

with ,   and .

(4) Update the auxiliary variable  by

with .

(5) Update the multipliers Λ and Γ by

(6) When satisfying the stopping condition, output  as the final thickness; otherwise, go to Step 1.

## Appendix B Algorithm 2: SPA with incomplete dictionary

(0) Initialization: compute the SVD of  as  Set , , ,  and . Set .

(1) Update the probe  as Step 1 of Algorithm 1.

(2) Update the thickness function  by

(3) Update  [imaginary unit ] with the real part updated by

with , and the imaginary part updated by

(4) Update the auxiliary variable  as Step 4 of Algorithm 1.

(5) Update the multipliers Λ and  by

(6) When satisfying the stopping condition, output  as the final thickness; otherwise, go to Step 1.
